# Supplementary material for: Association between oral microbiome and seven types of cancers in East Asian population: a two-sample Mendelian randomization analysis
Source: Front Mol Biosci. 2023 Nov 21;10:1327893. doi: 10.3389/fmolb.2023.1327893 (PMC10702768; doi:10.3389/fmolb.2023.1327893)
Supplement: Supplementary file 1 [file DataSheet1.ZIP › Supplementary Materials/MR plots of saliva/saliva═╝/ovarian cancer/pheno.3258_to_ovarian cancer_forest.pdf]

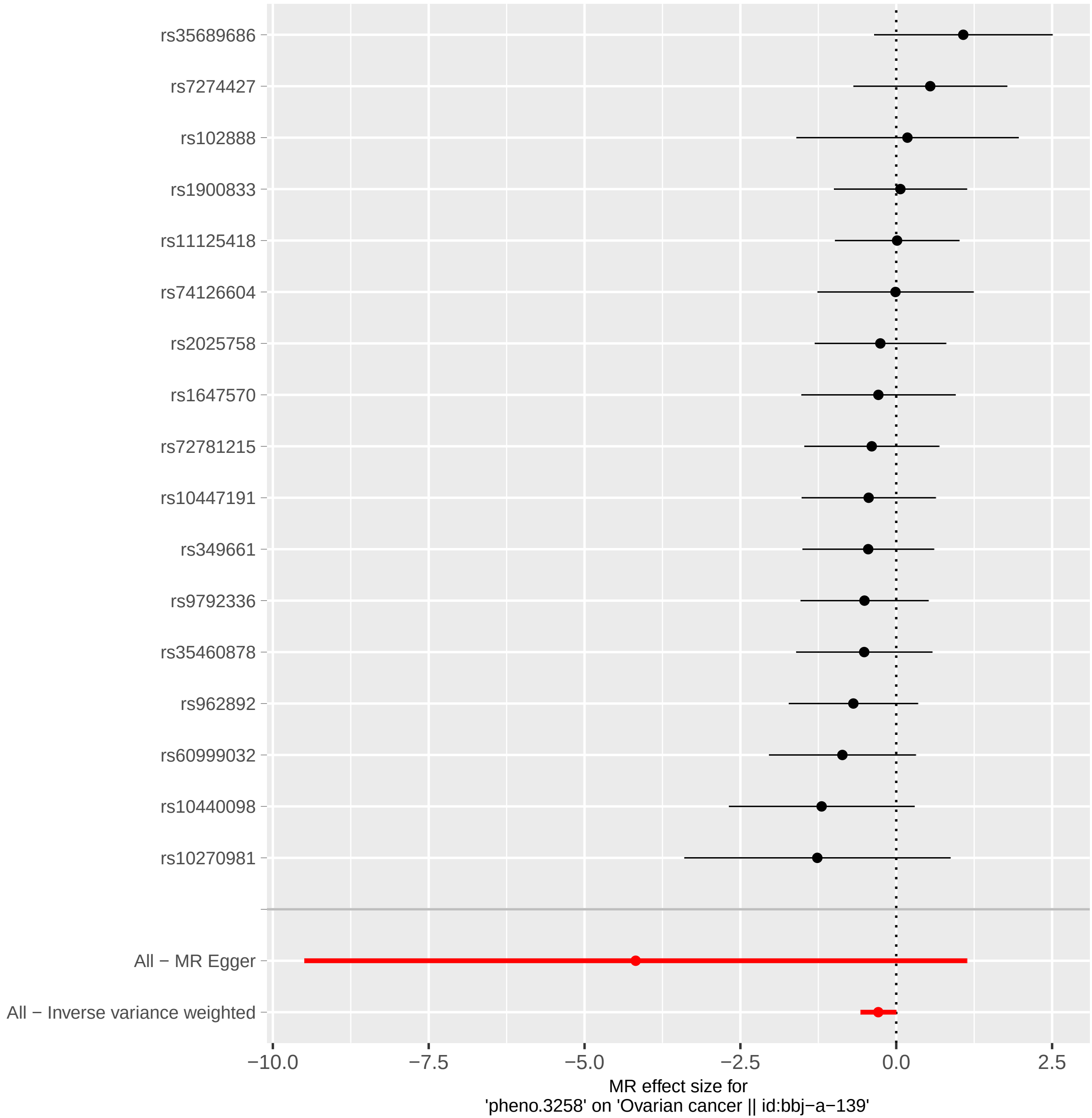
rs35689686
rs7274427
rs102888
rs1900833
rs11125418
rs74126604
rs2025758
rs1647570
rs72781215
rs10447191
rs349661
rs9792336
rs35460878
rs962892
rs60999032
rs10440098
rs10270981
All − MR Egger
All − Inverse variance weighted
−10.0
−7.5
−5.0
−2.5
0.0
2.5
MR effect size for
'pheno.3258' on 'Ovarian cancer || id:bbj−a−139'
